# *Etrog Citron* (*Citrus medica*) as a Novel Source of Antiviral Agents: Overview of Its Bioactive Phytochemicals and Delivery Approaches

**DOI:** 10.3390/pharmaceutics17091173

**Published:** 2025-09-09

**Authors:** Arik Dahan, Ludmila Yarmolinsky, Faina Nakonechny, Olga Semenova, Boris Khalfin, Sigal Fleisher-Berkovich, Shimon Ben-Shabat

**Affiliations:** 1 Department of Clinical Pharmacology, School of Pharmacy, Faculty of Health Sciences, Ben-Gurion University of the Negev, Beer-Sheva 8410501, Israel; yludmila@post.bgu.ac.il (L.Y.); boriskh83@gmail.com (B.K.); fleisher@bgu.ac.il (S.F.-B.); 2 Department of Chemical Engineering, Ariel University, Ariel 4070000, Israel; fainan@ariel.ac.il (F.N.); olga.semenova@msmail.ariel.ac.il (O.S.)

**Keywords:** etrog citron, *Citrus medica*, antiviral properties, flavonoids, terpenes, coumarins

## Abstract

The recent COVID-19 pandemic highlighted the significant challenge of insufficient antiviral pharmacological options. Edible plants offer a promising avenue for developing novel antiviral drugs. Etrog citron (*Citrus medica* L.), which is a valuable edible and medicinal plant, contains various antiviral phytochemicals, mainly flavonoids, coumarins, and terpenes. However, the therapeutic application of these compounds remains limited by factors such as poor solubility, limited bioavailability, and unclear mechanisms of action. The aim of the present article is to offer a comprehensive analysis of the antiviral phytochemicals extracted from various parts of *Citrus medica*, emphasizing their mode of action and delivery strategies that may allow turning these compounds into new antiviral drugs.

## 1. Introduction

Medicinal and edible plants and their constituents present a promising avenue for developing effective antiviral drugs [1,2,3,4]. All these plants deserve much attention because their active phytochemicals are not only potential antiviral drugs, but they also display a wide range of biomedical applications [5,6,7].

Etrog citron (*Citrus medica* L.) is a valuable edible and medicinal plant. We have recently thoroughly discussed its antimicrobial phytochemicals [8]. However, *C. medica* is not only an important source of antimicrobial compounds but also contains various antiviral phytochemicals as well [9]. Although the antiviral properties of this plant have been mentioned [10,11,12], an in-depth overview of the antiviral phytochemicals, their mechanisms of action, and strategies for their delivery is much needed. The antiviral phytochemicals of *C. medica* are of special interest because viral conditions remain a leading worldwide cause of morbidity and mortality [13]. In addition, the COVID-19 pandemic, caused by severe acute respiratory syndrome coronavirus 2 (SARS-CoV-2), impacted the everyday life of many people around the world because of historic numbers of cases and deaths [14]. Although vaccine development was successful, along with a high level of vaccination and reduction in transmission, SARS-CoV-2 tended to mutate, as has been noted with the South African variant (variant B.1.351), ‘Epsilon’ (B.1.429) in Taiwan, and ‘Mu’ (B.1.621) in Colombia [15].

Unfortunately, no vaccine or effective specific treatment is currently available in many viral infection cases; there are only some drugs for treating herpesviruses, influenza, hepatitis C, and HIV [16]. These drugs are costly and often ineffective because of viral resistance and adverse side effects. Therefore, naturally derived agents could serve as a promising alternative for treating viral infections. Plant-derived compounds represent broadly acting antivirals [17,18].

Knowledge of the molecular mechanisms of phytochemical antiviral actions is particularly important in planning an effective therapeutic approach. The application of antiviral compounds of *C. medica* in the pharmaceutical industry faces challenges such as low yield, solubility, and bioavailability. This review aims to highlight the most important antiviral compounds derived from various parts of *C. medica* L., along with their mechanisms of action and delivery strategies.

## 2. Methods

While several studies reported the chemical composition of *C. medica* L. [10,12,19], data on the antiviral phytochemicals are relatively scarce. A systematic, structured search of several electronic databases (PubMed, Google Scholar, Scopus, and Science Direct) was conducted using names of chemical compounds and widespread species of viruses. The multiple criteria sorting methods were applied [20]. We have extracted data regarding inclusion/exclusion criteria since 2002.

## 3. Antiviral Activity of Phytochemicals

*C. medica* L. has at least forty-seven morphotypes, which were determined after genetic analyses, with the chemical content of each morphotype varying widely [21]. Moreover, chemical content may change due to geography, climate, soil features, and so on [22]. We recently considered the most widespread phytochemicals in various morphotypes of *C. medica* [8]. Although the process of identifying active antiviral compounds in many morphotypes of this plant has not been finished yet, it is clear that all parts of *C. medica* L. have a high content of valuable phytochemicals with antiviral properties (Table 1, Table 2 and Table 3). The extracts, juices, and essential oils of *C. medica* contain various molecules, and their chemical complexity ensures the broad-spectrum modes of action and non-specific anti-viral features [23]. After being mixed in different proportions, they may produce novel solutions that can fight against ever-changing viruses in different modes. However, synergistic effects between compounds of *C. medica* were not reported.

Many antiviral flavonoids are present in various parts of *C. medica* (Table 1). The antiviral mechanisms by which these chemicals inhibit and act on viruses are shown in Table 1. It can therefore be concluded that the mode of inhibitory action for individual compounds may be associated with the type of virus and its structure. Based on the information contained in the current literature, the following antiviral mechanisms of flavonoids are known: inhibition of viral enzyme activities, obstructing attachment and entrance of viruses into cells, influence on different phases of viral DNA or RNA replication, and protein processing [24,25].

Quercetin is the most important representative of the flavonoids because it is widely distributed in all morphotypes of *C. medica* L. This flavonoid is able to inhibit various families of viruses; for example, *Flaviviridae*, *Herpesviridae*, *Orthomyxoviridae*, *Coronaviridae*, *Hepadnaviridae*, *Retroviridae*, *Picornaviridae*, *Pneumoviridae*, *Filoviridae*, and more [26]. Comparison of its chemical structure with several ligands associated with key SARS-CoV-2 proteins indicated that quercetin is similar to Remdesivir, the co-crystallized ligand of RNA-dependent RNA polymerase (RdRp); quercetin was more effective than Remdesivir with an IC50 of 1.149 μg/mL and the selectivity index (SI) 791 (Remdesivir with 9.54 μg/mL and SI: 6) [27].

Rutin (quercetin-3-rutinoside) is highly effective against HSV-1; it is interesting that its SI is 266, while the SI of its aglycons amounts only to 7.1 for this virus [28]. The chemical modifications of aglycone flavonoids to the glycosylated forms may enhance their antiviral activity [29].

Quercetin was tested in mice infected with the influenza virus; the level of superoxide radicals and lipid peroxidation products decreased significantly [30].

Although a review of thirteen randomized controlled clinical trials revealed quercetin’s therapeutic effects on COVID-19 infection [31], contradictory or inconsistent findings from different studies are present. The periods of intake and doses are not determined clearly; for example, the dose varies from 500 mg to 1500 mg in adults [31]. These discrepancies may be explained by the small sample size, the short duration of the treatment, the lack of laboratory tests, and the inclusion of only healthy people.

The low bioavailability of quercetin, its instability, and its ability to undergo glucuronidation, sulfation and methylation are well-known [32] and impede its antiviral potential [33]. For instance, synergistic effects of quercetin and murine alpha/beta interferon against Mengo virus infection were reported [34]. It is important to emphasize the great interest in coumarins as a scaffold for the synthesis of novel antiviral agents [35] due to a pharmacophore of a planar aromatic nucleus connected with a hydrogen bond acceptor and a lactone group as a facilitator of protein-ligand binding [36]. For example, a structure-activity relationships (SARs) study showed umbelliferone (Table 2) is a rich source of various derivatives with various biological activities, including antiviral ones [37]. The majority of antiviral coumarins were isolated from fresh fruits of *C. medica* (Table 2).

Antiviral terpenes of *C. medica* L. are interesting because of their high scientific value for the synthesis of new chemical entities [38] and their synergistic interaction with many active compounds [39]. As aromatic metabolites, they determine the flavor and fragrance of *C. medica* [40]. Many terpenes of *C. medica* are widely used in industry. For example, geraniol is present as an ingredient in 76% of twenty-one deodorants and 41% of fifty-nine domestic and household products [41,42]. Table 3 includes terpenes for which antiviral activity was investigated. However, many terpenes of *C. medica* L. were identified in the extracts of other plants as components of an antiviral mixture in which individual antiviral compounds were not identified.

Antiviral properties of carvacrol are attributed to its unique chemical structure, which is a combination of a hydrophobic aromatic ring with a hydrophilic phenolic hydroxyl group. Experiments in silico demonstrated the antiviral effect of carvacrol on SARS-CoV-2 associated with its high binding affinity towards the S1 SARS-CoV-2 spike protein [43]; 100% affinity of carvacrol was determined for molecular docking with one of the amino acid residues targeting the inhibition of the viral RNA polymerase [44]. In addition, carvacrol binds the SARS-CoV2- 3-chymotrypsin-like protease, which is important for the coronavirus replication cycle [45]. It was reported that the best poses of carvacrol docking simulations according to binding energy were from −7.9 to −3.5 (kcal/mol), and the docking score was −8.0 kcal/mol [46]. Carvacrol may target the SARS-CoV-2 spike protein of the Omicron variant RBD domain [47].

**Figure 1:**
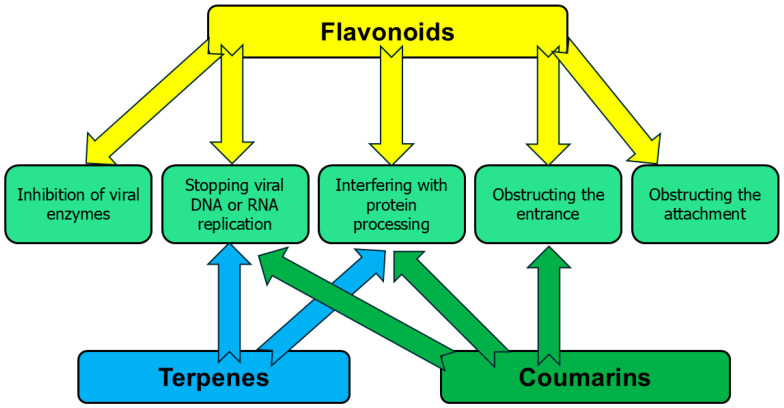
The mechanisms of antiviral activities of phytochemicals identified in *C. medica*.

Many phytochemicals of *C. medica* L. may have antiviral properties if they have antioxidant and anti-inflammatory properties [48]. In many cases, their antiviral activities were not investigated. For example, the antiviral properties of valencene [49], atalantaflavon [50], and many others were not evaluated, but their antioxidant and anti-inflammatory effects were reported. Figure 1 depicts the mechanisms underlying the antiviral activity of phytochemicals isolated from *C. medica*. Some studies were not adequately supported by cell culture assays and non-cell culture techniques [51] allowing to assess the mechanisms of action of active compounds against viruses; therefore, their modes of action remained unknown (Table 1, Table 2 and Table 3).

Chemical modifications of flavonoids, coumarins, and terpenes applying dimerization strategies may become one of the effective means to enhance their antiviral potential [52].

**Table 1:** Antiviral properties of the flavonoids identified in *C. medica*.

Compound	Structure	Part of the Plant	Virus	Mechanism	References
Apigenin	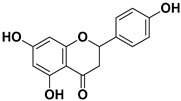	Leaves, flowers, mesocarp, endocarp	BPXV EBV HCV SARS-CoV-2	Inhibits synthesis of viral DNA, mRNA and proteins, Inhibition of HCV replication	[15,53,54,55]
Catechin	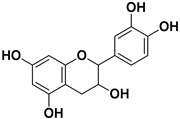	Mesocarp, endocarp, seeds, flavedo, pulp	Dengue virus Influenza A (H1N1)	Inhibit a post-entry stage of the DENV replication cycle. Inhibits neuraminidase	[56,57]
Caffeic acid	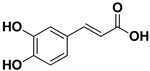	Exocarp, mesocarp, endocarp, seeds	Ilhéus virus Severe fever with thrombocytopenia syndrome (SFTS)	Interacts with the envelope Unknown	[58,59]
Chlorogenic acid	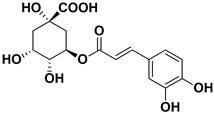	Endocarp, mesocarp	Infectious Bursal Disease Virus (IBDV)	Unknown	[60]
Dihydroquercetin	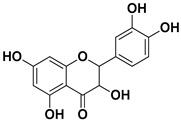	Exocarp, endocarp, seeds	Coxsackie B4 virus	Unknown	[61]
Diosmin	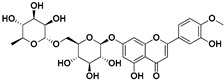	Flowers, leaves, mesocarp, endocarp	Influenza A, *Varicella zoster* virus (VZV)	Unknown	[62,63]
Epicatechin	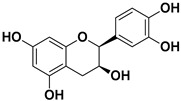	Flavedo, pulp	Singapore grouper iridovirus (SGIV) SARS-CoV-2	Impact on the replication of SGIV, Disrupts the interaction of ACE2 and SARS-CoV-2	[64,65]
Gallic acid	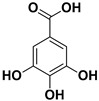	Exocarp, mesocarp, endocarp	Influenza A and B viruses	Unknown	[66]
Hesperidin	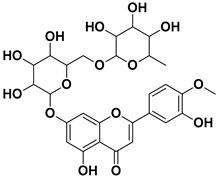	Flavedo, exocarp, endocarp, mesocarp, seeds, flowers, leaves	SARS-CoV-2	Inhibition of viral replication	[67]
Kaempferol 3-*O*-rutinoside	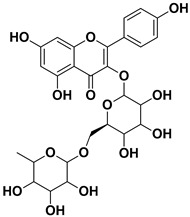	Flavedo	HSV-1, HSV-2 SARS-CoV-2	Unknown	[28,68]
Lonchocarpol A	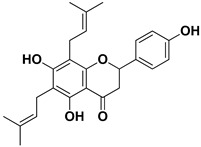	Root, stem	HIV	Unknown	[69]
Naringin	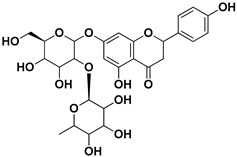	Fructus, exocarp, mesocarp, endocarp, seeds, flavedo	Chikungunya virus	Anti-nsP2 protease activity	[70]
Neodiosmin	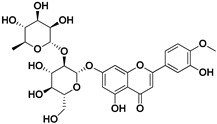	Fructus	SARS-CoV-2	Unknown	[71]
Nobiletin	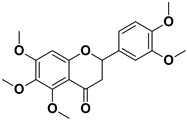	Exocarp, mesocarp, endocarp, seeds	Chikungunya virus	Translation/replication stages and viral entry	[72]
Quercetin	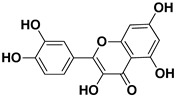	Flowers, leaves, mesocarp, endocarp	SARS-CoV-2 HCV	Suppresses the early phases of infection, interacts with viral replication proteases Inhibits NS3 protease activity	[73,74]
Rutin (quercetin-3-rutinoside)	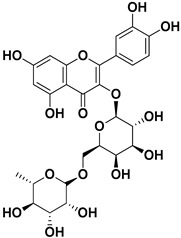	Flavedo	HSV-1, HSV-2	Unknown	[28]
Sakuranetin	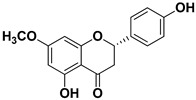	Leaves	Human rhinoviruses, Influenza B/Lee/40 virus	Unknown Inhibition of viral RNA synthesis	[75,76]
Salicylic acid	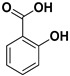	Fructus	HSV-1, H1N1 influenza A, adenovirus type 5	Unknown	[77]
Tangeritin	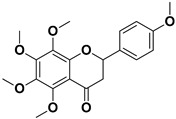	Exocarp, mesocarp, endocarp	RSV	Downregulates expression of RSV phosphoprotein	[78]
Vitexin	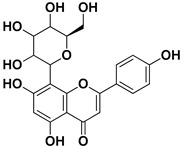	Exocarp, endocarp, seeds	Influenza A (H1N1) virus HSV-1 HAV	Inflammation reduction Inhibition of viral replication	[79]

**Table 2:** Antiviral properties of the coumarins identified in *C. medica*.

Compound	Structure	Part of the Plant	Virus	Mechanism	References
Citropten	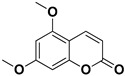	Stem, root, barks, fresh fruit	HIV-1	Unknown	[50]
Leptodactylone	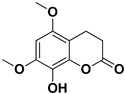	Fresh fruit	SARS-CoV-2	Unknown	[80]
Nordentatin	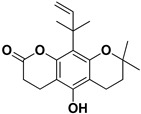	Fresh fruit	HIV-1, Hepatitis B	Unknown	[81,82]
Scopoletin	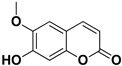	Fresh fruit, fructus	SARS-CoV-2	Inhibition of virus entry to the host cell	[83,84]
Umbelliferone	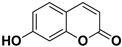	Fresh fruit	Viral hemorrhagic septicemia virus	Inhibition of VHSV replication, downregulation of Caspase 3 expression and inhibition of virus-induced apoptosis at later infection stages	[85]
Xanthyletin	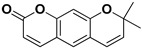	Stem, root, barks, fresh fruit	HIV-1	Unknown	[50]

**Table 3:** Antiviral properties of the terpenes identified in *C. medica*.

Compound	Structure	Part of the Plant	Virus	Mechanism	References
α-pinene	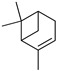	Flavedo	Bronchitis virus	Inhibition of nucleocapsid protein	[86]
α-terpineol	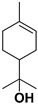	Flavedo, fresh fruits, mesocarp	HSV-1	Unknown	[87]
Carvacrol	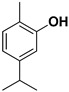	Flavedo, fresh fruits, mesocarp	HSV-2 Feline calcivirus, Murine norovirus	Inhibits the HSV-2 proliferation Unknown Unknown	[88,89]
Citral	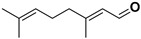	Fructus, flavedo, mesocarp	Yellow fever virus	Unknown	[90]
Citronellol	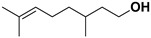	Flavedo	HSV-1	Unknown	[91]
Farnesol	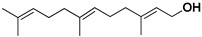	Flavedo	HSV-1	Unknown	[92]
Geraniol	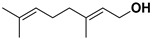	Fructus, flavedo	HSV-1	Changes the subcellular distribution of the oligonucleotides	[93]
Limonene	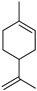	Flavedo, fructus, oil glands, exocarp, mesocarp, seeds	Drug-resistant influenza virus	Inhibition intracellular replication	[94]
Limonin	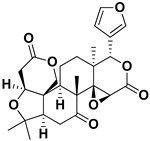	Citron waste, fructus, bark	HIV-1	Inhibition protease activity	[95]
Linalool	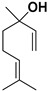	Flavedo	AVD-II	Unknown	[96]
Nomilin	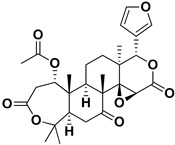	Fresh fructus	HIV-1	Inhibition protease activity	[95]
Obacunone	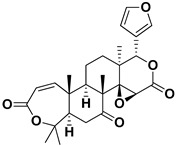	Fresh fructus	SARS-CoV-2	Disruption of target proteins	[97]

## 4. Applicability of Delivery Systems

**Figure 2:**
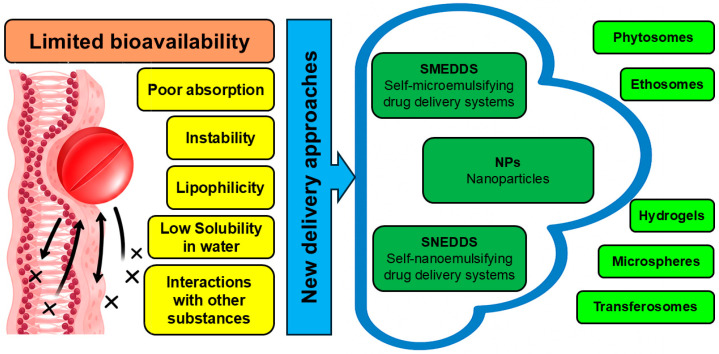
Delivery strategies that amplify the antiviral activity of phytochemicals identified in *C. medica*.

As mentioned above, all parts of *C. medica* are abundant with various antiviral chemicals, and these compounds are characterized by low water solubility and low bioavailability, which significantly limit their practical application. Although all these components are edible, their direct application with food significantly decreases their activity because proteins and lipids interact with them [98]. In addition, metabolism, chemical structures, chemical heterogeneity, and forms of intake are also responsible for affecting the bioavailability of antiviral phytochemicals. Modern nanotechnology tackles the above-mentioned problems by incorporating the antiviral phytochemicals of *C. medica* into various nanomaterials. Certain delivery approaches are recognized as capable to ensure optimal delivery of antiviral compounds with the abovementioned properties [8,13]. These include phytosomes, nanoparticles, self-microemulsifying drug delivery systems (SMEDDS), and self-nanoemulsifying drug delivery systems (SNEDDS hydrogels, microspheres, transferosomes and ethosomes) [13]. The bioavailability challenges and the available approaches to deal with them are summarized in Figure 2.

While the vast majority of the available delivery research studied isolated phytochemicals, one work investigated the fruit and leaf extract of *C. medica* within zinc oxide nanoparticles and demonstrated its effectiveness against the pathogenic avian influenza H5N1 virus [9]. Other studies associated with delivery systems were performed using antiviral phytocomponents derived from *C. medica.*

Phytosomes represent different forms of phospholipid-based delivery systems, combining either plant extracts or phytochemicals and phospholipids [99], with phosphate groups of phospholipids binding to hydroxyl parts of phytochemicals through H-bonds [100]. It is interesting that quercetin phytosomes increased the bioavailability rate of this compound twenty times in comparison with total quercetin in human plasma [101]. Furthermore, quercetin phytosomes were effective against SARS-CoV-2 viruses in the initial stages of COVID-19 infection [102]. Although phytosomes containing hesperidin enhanced solubility in a basic buffer system, there was excellent membrane permeation efficiency and better results in antioxidant activity than hesperidin [103]. Their antiviral properties were not investigated, and the antiviral phytosomal formulations were not released to the market because they were not developed deeply. In addition, their drawbacks include unstable nature, short life, and so on, which must be overcome. The electrosprayed nanoparticles in polyvinyl alcohol, which were loaded with chlorogenic acid, demonstrated significant antiviral activity against coronavirus (HCoV-229E) and Middle East respiratory syndrome coronavirus (MERS-CoV) and NRCEHKU270) [104].

In vitro experiments were performed with hesperidin in combination with favipiravir liposomal nanoformulations against SARS-CoV-2. As a result, this combination inhibited the replication of SARS-CoV-2 better than liposomes loaded by individual components or free favipiravir and hesperidin [105].

Metallic nanoparticles gained attention due to their contribution to targeted drug delivery [106]. These nanoparticles, containing silver, gold, zinc oxide, iron oxide, titanium dioxide, and so on, are effective antiviral agents without phytochemicals, depending on size ranges, morphologies, surface chemistry, and charges [107]. The possible antiviral mechanisms are associated with the attachment of nanoparticles to surface moieties of viruses with production of reactive oxygen species and the disruption of viral proteins [108]. Two antiviral terpenes of *C. medica*, carvacrol and geraniol, enhanced antiviral activity of nanoparticles of zinc oxide when several viruses were assessed, including SARS-Co-V-2 [109].

Silver nanoparticles with a mixture of flavonoids, gallic acid, chlorogenic acid, and naringenin significantly inhibited infectious laryngotracheitis virus and infectious bronchitis virus in chickens, with a possible mechanism of antiviral activity being interaction between the antiviral agent and the external viral envelope proteins [110].

Highly monodispersed gold nanoparticles, which were synthesized using gallic acid, were effective against two species of *Herpes simplex* virus due to blocking viral attachment and penetration into the host cells [111]. Antiviral effects of gold and silver nanoparticles with gallic acid against SARS-CoV-2 were presented in another research, with the presence of gallic acid decreasing the toxicity of nanoparticles [112].

It was reported that farnesol-containing nanoparticles caused inhibition of the spike proteins of SARS-CoV-2 by up to 83%, obstructing the attachment and entrance of viruses into the host cells due to the lipophilic structure of farnesol and its ability to interact with the double lipid layer of the viral envelope [113].

It is known that polymeric biomaterials are the most innovative and enticing possibility of delivery systems, especially biodegradable and biocompatible polymers. In fact, removal of the carrier when release of the drug occurs is an essential aspect of the system. The most widespread polymeric biomaterials for the formulation are polysaccharides, polypeptides, or phospholipids [114].

For example, chitosan is a cationic biocompatible polysaccharide [115]. It was reported that chitosan nanoparticles containing either gallic acid or quercetin significantly inhibited SARS-CoV-2 entry into the host cells [116].

Gallic acid was successfully transformed into biocompatible graphene quantum dots which enhanced antiviral activity against pseudorabies virus both in vitro and in vivo, inhibiting the viral adsorption, invasion and replication [117].

The antiviral compounds of *Citrus medica* are similar to other edible plants; it has been proposed that they are likely to have minimal toxicity. Indeed, the toxicity of nanocarriers in any shape or form needs additional research.

For the clinical use of *C. medica* nanotechnological products, it is necessary to study their interaction with human cells, tissues, and organs following long-term administration courses. Thus, in vitro and in vivo studies for the potential cytotoxic impact of these products are required prior to clinical trials.

Although laboratory studies in cell models show antiviral activity of active compounds of *C. medica* at certain concentrations (e.g., 20–50 µM), embodiment of these findings into human therapy requires thorough pharmacokinetic estimations. Due to their low bioavailability, such concentrations of flavonoids, coumarins and terpenes are often unrealistic through oral administration. Indeed, without advanced delivery systems or chemical modifications, these treatment concentrations may not be clinically realistic.

**Figure 3:**
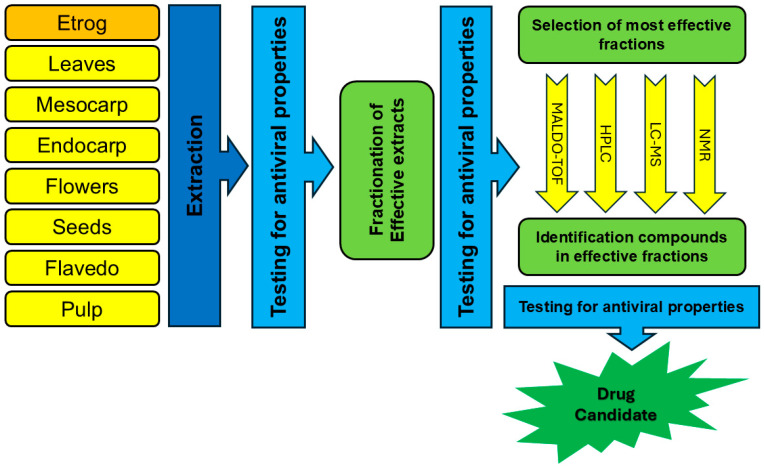
Roadmap for development of *C. medica*-derived antivirals.

As described throughout this article, the clinical translation of active compounds derived from *Citrus medica* is impeded by challenges such as an incomplete understanding of antiviral mechanisms, and the need for comprehensive pharmacokinetic modeling long-term safety evaluation, and large-scale clinical validation. Our proposed roadmap for the development of *Citrus medica*-derived antivirals is depicted in Figure 3.

## 5. Conclusions

Accumulating data shows that phytochemicals of *C. medica* could become valuable antiviral material. Unfortunately, the chemical compositions of many varieties of etrog citron are unknown, and many important antiviral compounds have yet to be identified and estimated. Figure 3 represents a possible roadmap for the development of *C. medica*-derived antivirals. Comparative metabolomics methods may highlight a wide range of secondary metabolites. In addition, SAR-guided phytochemical modifications are effective means to enhance antiviral properties of flavonoids, coumarins, and terpenes of *C. medica*.

Preclinical studies in animal models and clinical trials were almost not performed using antiviral compounds of *C. medica*. Validation studies in vivo are very important to test antiviral compounds, extracts, juices, and essential oils of *C. medica*, providing crucial data on pharmacokinetics and pharmacodynamics in a comprehensive way.

The broad use of antiviral compounds of *C. medica* has been hampered by its low water solubility and low bioavailability. Emerging studies have suggested that phytosomes, nanoparticles, self-microemulsifying drug delivery systems (SMEDDS), and self-nanoemulsifying drug delivery systems (SNEDDS hydrogels, microspheres, transferosomes, and ethosomes) may enhance the solubility and physiological efficacy of antiviral compounds of *C. medica.* Appropriate nanocarriers can amplify the antiviral efficacy of active compounds derived from *C. medica*.

Despite the growing interest in nanotechnology and natural products, major pharmaceutical companies are generally reluctant to invest in these areas. As a result, the development of innovative antiviral products in this field is largely driven by start-ups and small pharmaceutical companies, which often face significant challenges in securing investment [118]. We hope that our proposed roadmap for the development of *Citrus medica*-derived antivirals (presented in Figure 3) will aid in overcoming these challenges.
